# A novel variant of *DNM1L* expanding the clinical phenotypic spectrum: a case report and literature review

**DOI:** 10.1186/s12887-023-04442-y

**Published:** 2024-02-10

**Authors:** Zhenkun Zhang, Xiaofan Bie, Zhehui Chen, Jing Liu, Zhenhua Xie, Xian Li, Mengjun Xiao, Qiang Zhang, Yaodong Zhang, Yanling Yang, Dongxiao Li

**Affiliations:** 1 Henan Provincial Clinical Research Center for Pediatric Diseases, Henan Children’s Neurodevelopment Engineering Research Center, Children’s Hospital Affiliated to Zhengzhou University, Zhengzhou, 450018 China; 2 Department of Pediatrics, Peking University First Hospital, Beijing, 100034 China

**Keywords:** *DNM1L* gene, Mitochondrial diseases, Nonsense variant, Phenotypic spectrum

## Abstract

**Background:**

Mitochondrial diseases are heterogeneous in terms of clinical manifestations and genetic characteristics. The dynamin 1-like gene (*DNM1L*) encodes dynamin-related protein 1 (DRP1), a member of the GTPases dynamin superfamily responsible for mitochondrial and peroxisomal fission. *DNM1L* variants can lead to mitochondrial fission dysfunction.

**Case presentation:**

Herein, we report a distinctive clinical phenotype associated with a novel variant of *DNM1L* and review the relevant literature. A 5-year-old girl presented with paroxysmal hemiplegia, astigmatism, and strabismus. Levocarnitine and coenzyme Q_10_ supplement showed good efficacy. Based on the patient’s clinical data, trio whole-exome sequencing (trio-WES) and mtDNA sequencing were performed to identify the potential causative genes, and Sanger sequencing was used to validate the specific variation in the proband and her family members. The results showed a novel *de novo* heterozygous nonsense variant in exon 20 of the *DNM1L* gene, c.2161C>T, p.Gln721Ter, which is predicted to be a pathogenic variant according to the ACMG guidelines. The proband has a previously undescribed clinical manifestation, namely hemiparesis, which may be an additional feature of the growing phenotypic spectrum of *DNM1L*-related diseases.

**Conclusion:**

Our findings elucidate a novel variant in *DNM1L*-related disease and reveal an expanding phenotypic spectrum associated with *DNM1L* variants. This report highlights the necessity of next generation sequencing for early diagnosis of patients, and that further clinical phenotypic and genotypic analysis may help to improve the understanding of *DNM1L*-related diseases.

**Supplementary Information:**

The online version contains supplementary material available at 10.1186/s12887-023-04442-y.

## Background

Mitochondria are dynamic organelles that undergo constant fusion and fission, both of which are mediated by nuclear DNA (nDNA)-encoded proteins that act on the mitochondrial membrane [1]. A proper dynamic balance between mitochondrial fission and fusion is essential to maintain the morphology of the mitochondrial network and function. Defects in mitochondrial proteins can lead to abnormal fusion and fission, resulting in clinical disease [2]. In mammals, DRP1, a protein encoded by the *DNM1L* gene, is a core component of the mitochondrial fission process and contains an N-terminal GTPase domain, a middle domain, a non-conserved variable domain (VD), and a C-terminal GTPase effector domain (GED) [3–5]. The major functional units are the GTPase domain, which provides the mechanical force for membrane contraction, and the middle domain, which mediates DRP1 oligomerization [6]. In addition, DRP1 is also involved in mediating peroxisomal fission [7].

DRP1 impairment causes two neurological diseases associated with pathogenic variants in *DNM1L* (MIM*603,850), including encephalopathy due to defective mitochondrial and peroxisomal fission-1 (EMPF1, MIM #614,388) and optic atrophy 5 (MIM#610,708). EMPF1, caused by monoallelic or biallelic pathogenic *DNM1L* variants, is frequently associated with developmental delay, hypotonia, refractory epilepsy, and even death [8–10]. The first reported *de novo* heterozygous *DNM1L* variant, c.1184C>A, p.Ala395Asp, caused a disease phenotype manifested by global developmental delay, neonatal encephalopathy, microcephaly, and optic atrophy [10]. The patient died suddenly at 37 days of age with minimal developmental progress. It has been reported that the p.Ala395Asp variant is located in the middle domain of DRP1 and impairs higher order assembly and GTPase activity of DRP1, resulting in abnormal mitochondrial and peroxisomal fission [11]. Recently, an increasing number of heterozygous variants in the DRP1 middle domain have been reported to cause severe encephalopathy with epilepsy and/or failure to thrive [12–14]. In contrast, heterozygous variants in the GTPase domain appear to be associated with a milder phenotype limited to isolated optic atrophy [15]. To our knowledge, disease-causing variants in the GED have rarely been reported in humans.

Here, we describe a 5-year-old girl with a novel c.2161C>T, p.Gln721Ter *DNM1L* variant identified in the GED. She had a different clinical presentation compared to previously reported cases, expanding the clinical presentation of *DNM1L*-related mitochondrial disease. Furthermore, due to the lack of studies on the phenotype-genotype correlation of *DNM1L*-related diseases, a review of the literature summarizing the relevance of clinical and genetic features may deepen the understanding of the disease.

## Case presentation

### Methods

#### Literature acquisition

The clinical data of the proband were collected from December 2020 to June 2023 in the Children’s Hospital Affiliated to Zhengzhou University, China. Furthermore, we conducted a literature review via PubMed (up to June 2023), using the following search string: “mitochondrial diseases” or “DRP1” or “EMPF1” associated to the keyword “*DNM1L*” and “children”. Additionally, a manual reference check of the retrieved literature was performed.

#### Whole-exome sequencing and mtDNA sequencing

This study was approved by the Ethics Committee of Henan Children’s Hospital. After obtaining informed consent, peripheral blood samples were collected from the proband and her family members including parents and sister. Genomic DNA was extracted from 200 µL of peripheral blood from each participant using the Qiagen DNA Blood Midi/Mini kit (Qiagen GmbH, Hilden, Germany) and sheared to approximately 200 bp. The DNA fragments were hybridized and captured by NanoWES according to the manufacturer’s protocol. The libraries were then quantified by qPCR and the size distribution was determined using an Agilent Bioanalyzer 2100 (Agilent Technologies, Santa Clara, CA, USA). The trio-WES was performed using the Illumina HiSeq2000 sequencer platform (Illumina, San Diego, CA, USA) with 150 bp pair-end sequencing mode. Raw data were obtained after processing with CASAVA v1.82 software (Illumina, San Diego, CA, USA). Sequencing reads were aligned to the human reference genome (hg19/GRCh37) using BWA tool (v0.7.12r1044; http://biobwa.sourceforge.net/) and PCR duplicates were removed by using Picard v1.57 software (http://picard.sourceforge.net/). In general, the test platform detected more than 95% of the target regions with a sensitivity of >99%. Verita Trekker® Variants Detection System (BerryGenomics, Beijing, China) and GATK software (https://software.broadinstitute.org/gatk/) were used for variant calling, followed by ANNOVAR software and Enliven® Variants Annotation Interpretation System (BerryGenomics, Beijing, China) for annotation. In addition, suspicious variants were evaluated according to the variation interpretation guidelines of American College of Medical Genetics and Genomics (ACMG) [16].

### Case report and results

**Fig. 1:**
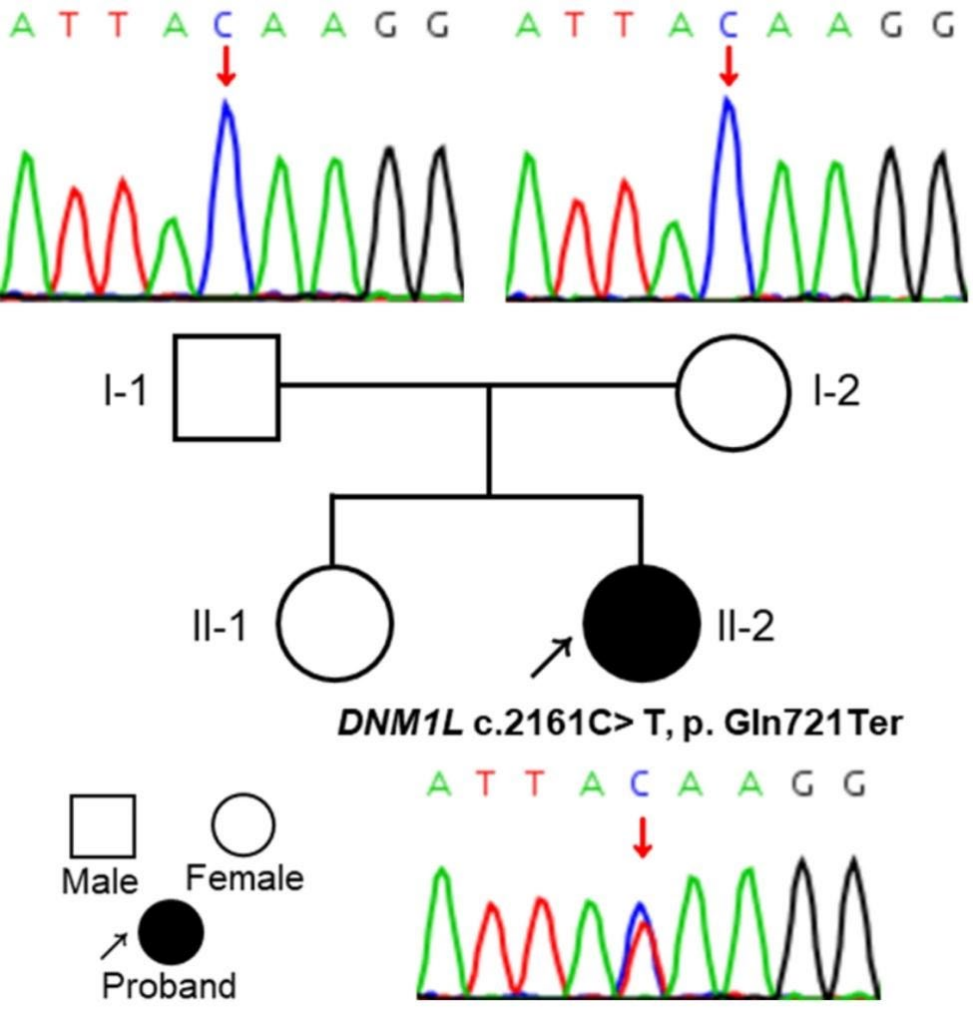
Family pedigree and results of the variant in the *DNM1L* gene of the proband (II-2). Sanger sequencing confirmed the presence of the heterozygous variant c.2161C>T, p.Gln721Ter in the proband compared to the wild type sequence in the parents

The case is the second child from non-consanguineous Chinese parents, born at term with normal birth parameters and has a healthy older sister. The family pedigree is shown in Fig. 1. She developed normally from the neonatal period until 1.5 years, apart from presenting with astigmatism and strabismus. She was able to call Papa and Mama at 10 months of age, had normal intelligence and was walking at 14 months. At the age of 1.5 years, she was found to have left-sided limb dysfunction preceded by a febrile illness. As the patient had left lower extremity weakness, she had left tip toe walking. The episodic limb dysfunction occurred every 1 to 3 weeks and lasted from 5 min to about 1 day. She was physically immobile during severe episodes, but her symptoms would be relieved after waking up the next day. The girl also had right-sided limb weakness with a right-sided hemiplegic gait and was suspected of having alternating hemiplegia of childhood (AHC). However, sequencing of AHC-related genes showed no significant abnormalities, such as the *ATP1A3* gene and the *ATP1A2* gene.

Physical examinations of the patient were unremarkable, except for strabismus. Extensive workups were performed for potential etiology including a normal blood ammonia level, lactate level, pyruvate level, serum amino acids, acylcarnitine profiles, liver and kidney function, electromyography (EMG), and electroencephalogram (EEG). Urinary organic acid results were unremarkable. Magnetic resonance imaging (MRI) of the brain at the age of 3 years showed no significant abnormalities. The patient was reported to have hemiparesis preceded by febrile illness or excessive exercise, but long-term treatment with levocarnitine (1 g/d) and coenzyme Q_10_ (20 mg/d) was started 6 months after the first episode of hemiparesis. After 2 months of treatment with coenzyme Q_10_ and levocarnitine, she has had no further episodes of hemiplegia and motor function had returned to normal. The patient is now 5 years old, with a weight of 16 kg, a height of 102 cm, a head circumference of 48.5 cm, and is doing well at school.

**Table 1:** Prediction of pathogenicity of the identified heterozygous variant c.2161C>T, p. Gln721Ter in the *DNM1L* gene

Gene	variant	Mutation Taster		FATHMM_MKL		CADD_Phred	
		Score	Prediction	Score	Prediction	Score	Prediction
*DNM1L* (NM_012062.5)	c.2161C>T, p.Gln721Ter	0.999	D	0.992	D	46	D

*D* Damaging

The trio-WES was used to identify potentially pathogenic genetic variants and Sanger sequencing was applied to confirm the identified variants. The results of the Sanger sequencing were analyzed using Chromas software, while the possible pathogenic effects of variants were predicted as previously described [17]. Furthermore, sequencing of the entire mtDNA genome of the child showed no pathologically significant variants. We identified a heterozygous stop-gain mutation of the *DNM1L* gene in the proband: c.2161C>T (exon 20, NM_012062.5, chr12:32896294), resulting in a stop-gain mutation NP_036192.2: p.Gln721Ter. The results of Sanger sequencing confirmed its absence in her parents, suggesting that the variant is *de novo* (Fig. 1). The variant was not found in human disease databases (including the 1000 Genomes Project, EXAC, gnomAD, and dbSNP databases), and was predicted to be disease-causing by online bioinformatics tools (Table 1).

**Fig. 2:**
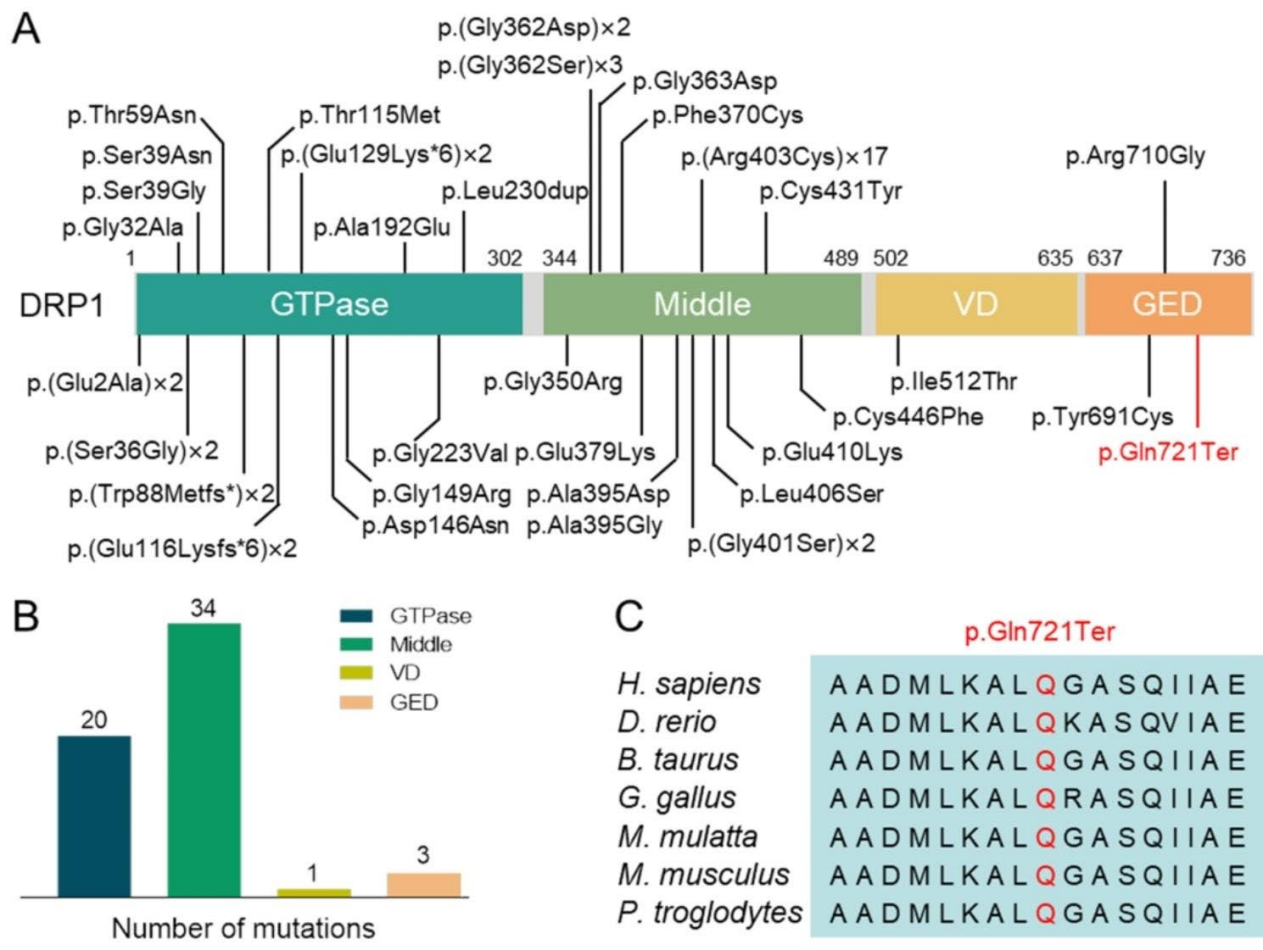
Genetic location of *DNM1L* gene variants associated with mitochondrial diseases identified to date. **A** Schematic representation of the location of identified variants in each domain of the DRP1 protein. Changes in amino acid distribution associated with protein domains. Variants reported in this study are shown in red, and “x” indicates the number of cases. **B** The number of previously reported variants in each domain. **C** Comparison of the amino acid sequences of the DRP1 protein in different species. The amino acid sequence of the variant site is highly conserved (red font)

*DNM1L* c.2161C>T, p.Gln721Ter was a nonsense mutation and was classified as likely pathogenic: PVS1_Moderate + PS2_Moderate + PM2_supporting according to the ACMG guidelines. Other previously reported pathogenic variants were then used for comparison, as described in Fig. 2a. The identified amino acid was highly conserved across different species as shown in Fig. 2c. No other suspected variants were found in the known disease-causing genes that might explain the clinical phenotype of our patients (Supplementary Table 1).

## Review of the literature

Based on the differences in phenotypes observed in the patients studied, all reported cases of *DNM1L* variants were reviewed (Supplementary Table 2) [5, 8–10, 12–15, 18–40]. A total of 53 cases were analyzed together with our case. In the described cases, the median age at which patients initially presented with clinical manifestations of specific organs or systems due to *DNM1L* variants was 12 months (range, neonatal period to 13 years). 40.0% of the cases eventually died (range, 8 days to 20 years), with a minimum age of death of 8 days and an average age of death of 5.7 years.

The phenotypic spectrum of *DNM1L* variants included neurological, craniofacial, cardiac, and ocular features. Neurological manifestations such as developmental delay/regression, hypotonia, ataxia, peripheral neuropathy, and epilepsy were the main characteristic features of patients with EMPF1. Of the 50 reported cases (except for pedigrees 1, 2, and 14), clinical manifestations included developmental delay/regression (DD and DR) in 74.0% (37/50), epilepsy (GTCS, SRSE, RSE, and FSE) in 68.0% (34/50), and dystonia in 32.0% (16/50). Other symptoms included 9 cases of peripheral neuropathy, 9 cases of microcephaly, 9 cases of encephalopathy, 8 cases of ataxia, 6 cases of pain insensitivity, 6 cases of dysarthria, and 5 cases of strabismus (Table 2). Regarding the auxiliary examinations, 68.0% (34/50) of the cases showed abnormal brain MRI and 44.0% (22/50) of the cases showed abnormal EEG. In addition, 42.0% (21/50) of the cases had elevated lactate.

To date, 33 pathogenic and/or potentially pathogenic variants have been reported in patients. *De novo* variants were identified in 79.2% (42/53) of the cases, and homozygous variants were identified in only 3.8% (2/53) of cases [5, 9]. As shown in Fig. 2b, more than half of the variants were located in the middle domain of the DRP1 protein, and 67.6% (23/34) of the middle domain variants occurred mainly in exon 11, confirming that exon 11 was the hotspot of variants in the whole *DNM1L* gene.

**Table 2:** Summary of key clinical features in patients with *DNM1L* variants in different domains

**Clinical data** **% (n/n) or n**	**GTPase domain ** **(n = 13)** ^**a**^	**Middle domain** **(n = 34)** ^**b**^	**GTPase effector domain** **(n = 3)**	**Total morbidity** **% (n/N)**		
**Age of onset (year)**	0.25 (0–6)	2.4 (0–13)	1.5 (0.25–3)			
**Sex**						
Male	46.2% (6/13)	52.9% (18/34)	33.3% (1/3)	50.0% (25/50)		
Female	53.8% (7/13)	47.1% (16/34)	66.7% (2/3)	50.0% (25/50)		
**Outcome**						
Alive	76.9% (10/13)	52.9% (18/34)	66.7% (2/3)	60.0% (30/50)		
Died	23.1% (3/13)	47.1% (16/34)	33.3% (1/3)	40.0% (20/50)		
**Abnormal development** ^**c**^				**74.0% (37/50)**		
Developmental delay	61.5% (8/13)	70.6% (24/34)	33.3% (1/3)	66.0% (33/50)		
Developmental regression	0	17.6% (6/34)	33.3% (1/3)	14.0% (7/50)		
**Epilepsy** ^**d**^				**68.0% (34/50)**		
Generalized tonic-clonic seizures	23.1% (3/13)	26.5% (9/34)	0	24.0% (12/50)		
Super refractory status epilepticus	7.7% (1/13)	17.6% (6/34)	0	14.0% (7/50)		
Refractory status epilepticus	0	14.7% (5/34)	0	10.0% (5/50)		
Focal status epilepticus	0	26.5% (9/34)	0	18.0% (9/50)		
**Dystonia**	38.5% (5/13)	32.4% (11/34)	0	**32.0% (16/50)**		
**Peripheral neuropathy**	53.8% (7/13)	2.9% (1/34)	33.3% (1/3)	**18.0% (9/50)**		
**Microcephaly**	15.4% (2/13)	20.6% (7/34)	0	**18.0% (9/50)**		
**Encephalopathy**	15.4% (2/13)	17.6% (6/34)	33.3% (1/3)	**18.0% (9/50)**		
**Ataxia**	46.2% (6/13)	5.9% (2/34)	0	**16.0% (8/50)**		
**Pain insensitivity**	15.4% (2/13)	11.8% (4/34)	0	**12.0% (6/50)**		
**Dysarthria**	38.5% (5/13)	2.9% (1/34)	0	**12.0% (6/50)**		
**Strabismus**	15.4% (2/13)	5.9% (2/34)	33.3% (1/3)	**10.0% (5/50)**		
**Abnormal brain MRI**	38.5% (5/13)	82.4% (28/34)	33.3% (1/3)	**68.0% (34/50)**		
**Abnormal EEG**	15.4% (2/13)	58.8% (20/34)	0	**44.0% (22/50)**		
**Increased lactate level**	46.2% (6/13)	47.1% (16/34)	0	**42.0% (21/50)**		
***De novo*** **variants (N = 53)**	72.7% (8/16)	91.2% (31/34)	100% (3/3)	**79.2% (42/53)**		

Data are shown as prevalence % (n/n) or n (number).

^a^The included patients were excluded from pedigrees 1, 2, and 14.

^b^The included patients contained patient 22, whose two variant sites were located in the middle and variable domain, respectively.

^c^Count only once when two of the following phenotypes are present at the same time.

^d^Count only once when two or more of the following phenotypes are present at the same time.

## Discussion and conclusions

In recent years, mitochondrial genes and nuclear genes related to mitochondrial structure and function have been extensively identified. *DNM1L*, encoding DRP1, is a member of the dynamin superfamily of GTPases that mediates mitochondrial and peroxisomal fission [3, 7]. Currently, mitochondrial diseases associated with *DNM1L* are rarely studied. The first case of *DNM1L* deficiency was reported in 2007 by Waterham et al., who described a case of infanthood-onset of mitochondrial disease with global developmental delay, microcephaly, abnormal brain development, optic atrophy, and persistent hyperlactatemia [10]. Subsequent studies have further broadened the clinical phenotypic spectrum.

The pathogenic variant in *DNM1L* is associated with a neurological disorder called “EMPF1” and has been described as a fatal encephalopathy. Affected individuals have different phenotypes ranging from severe hypotonia leading to death in the neonatal period to developmental delay/regression (DD/DR), with or without epilepsy [18, 23, 26]. Interestingly, the *DNM1L* variant in pedigrees 1, 2, and 14 exhibited a milder phenotype, familial isolated optic atrophy, and the results of functional analysis of these variants suggested a dominant negative mechanism [15, 41]. Our patient had an onset at 1.5 years of age and presented with paroxysmal limb motor dysfunction, manifested by unilateral limb weakness, tiptoeing, and a hemiplegic gait on the affected side. This condition has not been seen in previous reports, revealing an extended phenotype of the *DNM1L* variant. Interestingly, treatment with coenzyme Q_10_ and Levocarnitine was effective, contrary to reported cases [14, 19, 22, 33]. Furthermore, she was found to have strabismus and visual impairment, consistent with the phenotype of the novel variant of the *DNM1L* gene identified [15, 19], but without epilepsy throughout the developmental milestones.

Generally, pathogenic variants involving the DRP1 middle domain also appear to be more severe than those affecting the GTPase domain, which mainly manifest as visual abnormalities with or without a tendency to neurological and developmental deficits [5, 8, 15, 24]. Variants in the GTPase domain have been reported in 29 patients, all of whom had a relatively stable course, except for two who developed respiratory failure and died soon after birth [23]. Moreover, we found that the incidence of developmental delay/regression and epilepsy was greater in the middle domain than that in the GTPase domain, and the proportion of epileptic persistence and refractory epilepsy was similarly higher in the middle domain (Table 2). The reason may be that variants in the GTPase domain may disrupt the binding of the GTPase domain, and the presence of multiple interaction sites in the oligomers may hinder their cohesion, resulting in a relatively mild clinical phenotype [5].

The GED plays a role in the activation of the GTPase domain. Previous studies have shown that the GED is involved in the formation and stability of DRP1 homodimeric complexes and that the corresponding GED variant in human DRP1 reduces GTPase activity and decreases intramolecular interactions between the GED and the middle domain [8, 40, 42]. The first patient with the GED variant (p.Tyr691Cys) was found to have neonatal hypotonia that progressed to unprovoked myoclonic seizures, developmental delay, and static encephalopathy accompanied by nystagmus, optic atrophy, and scoliosis. The second case of the GED variant (p.Arg710Gly) showed similar symptoms to those of the p.Tyr691Cys variant. Interestingly, both patients with GED variants exhibited less severe phenotypes such as epilepsy, optic atrophy, and impaired mobility [8, 40]. Here, we describe a patient with a distinct phenotype due to a novel variant in the *DNM1L* gene located in the GED of the DRP1 protein. As far as we know, this is the third GED variant identified to date. The variant produces a stop-gain mutation (p.Gln721Ter) in the DRP1 protein, resulting in an early termination codon at position 721. Our patient showed no epilepsy or optic atrophy throughout the developmental milestones except for hemiplegia and strabismus, and had the mildest phenotype compared to the patients mentioned above. However, as the interactions, functional and structural roles of the GED of mammalian DRP1 remain unclear and reports of GED variants associated with human disease are lacking, the impact of this domain remains to be further explored. Additionally, based on the difference in age of onset, whether the child eventually presented with epilepsy or encephalopathy needs to be considered in follow-up.

In conclusion, we identified a novel *de novo* heterozygous *DNM1L* variant in one individual in a Chinese family. Not only did the case share similar clinical features compared with previous cases, but the newly reported manifestations, such as hemiparesis, broadened the phenotypic spectrum of the *DNM1L* variant. *DNM1L*-related diseases have heterogeneous clinical manifestations and genetic features that may affect multiple systems, but the diagnosis of this disease requires genetic testing for confirmation. Furthermore, future functional studies will be essential to confirm that the variant is truly deleterious. Our systematic description of the clinical presentation and progression of patients will help to identify other rare patients with *DNM1L* variants in a timely manner. Importantly, this report provides a better understanding of the impact of *DNM1L* variants on phenotypic outcomes, which will inform the clinical diagnosis of *DNM1L*-related diseases.

### Electronic supplementary material

Below is the link to the electronic supplementary material.

Supplementary Material 1

## Data Availability

The original contributions presented in the study are included in the article and further inquiries can be directed to the corresponding author.

## Abbreviations

ACMG: American College of Medical Genetics and Genomics
*DNM1L*: Dynamin 1-like
DRP1: Dynamin-related protein 1
DD: Developmental delay
DR: Developmental regression
EMPF1: Encephalopathy due to defective mitochondrial and peroxisomal fission-1
FSE: Focal status epilepticus
GED: GTPase effector domain
GDD: Global developmental delay
GTCS: Generalized tonic-clonic seizures
MRI: Magnetic resonance imaging
N/A: Not applicable
ND: No description
nDNA: Nuclear DNA
RSE: Refractory status epilepticus
SRSE: Super refractory status epilepticus
trio-WES: Trio-whole-exome sequencing
VD: Variable domain
