# Supplementary material for: A novel variant of DNM1L expanding the clinical phenotypic spectrum: a case report and literature review
Source: BMC Pediatr. 2024 Feb 10;24:104. doi: 10.1186/s12887-023-04442-y (PMC10858475; doi:10.1186/s12887-023-04442-y)
Supplement: Supplementary file 1 — Supplementary Material 1 [file 12887_2023_4442_MOESM1_ESM.docx]

*Supplementary Data*

**A novel variant of *DNM1L* expanding the clinical phenotypic spectrum: a case report and literature review**

Zhenkun Zhang#^1^, Xiaofan Bie#^1^, Zhehui Chen^2^, Jing Liu^1^, Zhenhua Xie^1^, Xian Li^1^, Mengjun Xiao^1^, Qiang Zhang^1^, Yaodong Zhang^1^, Yanling Yang*^2^, Dongxiao Li*^1^

^1^Henan Provincial Clinical Research Center for Pediatric Diseases, Henan Children's Neurodevelopment Engineering Research Center, Children's Hospital Affiliated to Zhengzhou University, Zhengzhou, 450018, China.

^2^ Department of Pediatrics, Peking University First Hospital, Beijing, 100034, China.

# The authors contribute equally to the manuscript.

Corresponding author:

Dongxiao Li, Email: [li_dongxiao@sina.com](mailto:li_dongxiao@sina.com);

Yanling Yang, Email: [organic.acid@vip.126.com](mailto:organic.acid@vip.126.com)

| **Supplementary Table 1**   \| **Supplementary Table 1** Other variants of unknown significance detected in the patient’s whole-exome sequencing \| \| \| \| \| \| \| \|  \| \| --- \| --- \| --- \| --- \| --- \| --- \| --- \| --- \| --- \| \| **Gene** \| **Chromosome** \| **Variant** \| **Variant type** \| **Variant loci** \| **Genotype** \| **dbSNP/ dbVar ID** \| **Inheritance** \| **Variant  classification** \| \| *OPA1* \| 3 \| c.2348A>G, p.Asn783Ser \| Missense \| Exon 24 \| Het \| rs749143995 \| Maternal \| VUS \| \| *AFF2* \| X \| c.1084C>T, p.Arg362Trp \| Missense \| Exon 4 \| Het \| rs782691184 \| Maternal \| VUS \| \| *KIF5A* \| 12 \| c.65C>T,  p.Ala22Val \| Missense \| Exon 1 \| Het \| N/A \| Maternal \| VUS \| \| *ADCY6* \| 12 \| c.2783T>A, p.Leu928Gln \| Missense \| Exon 17 \| Het \| N/A \| Maternal \| VUS \| \| Abbreviations: Het = heterozygous; N/A = not applicable; VUS = variant of uncertain significance \| \| \| \| \| \| \| \|  \|   **Supplementary Table 2**    **Supplementary Table 2** **Variable Clinical Characteristics in Children With *DNM1L* Variants** | | | | | | | |  |
| --- | --- | --- | --- | --- | --- | --- | --- | --- | --- | --- | --- | --- | --- | --- | --- | --- | --- | --- | --- | --- | --- | --- | --- | --- | --- | --- | --- | --- | --- | --- | --- | --- | --- | --- | --- | --- | --- | --- | --- | --- | --- | --- | --- | --- | --- | --- | --- | --- | --- | --- | --- | --- | --- | --- | --- | --- | --- | --- | --- | --- | --- | --- | --- | --- | --- | --- | --- | --- | --- | --- | --- |
| **Patients  (sex)** | **Age at onset** | **Clinical features and relevant biochemical findings** | **Clinical course and age at assessment** | **Variant** | **Variant loci**  **and domain** | **Inheritance** | **Reference** | |
| P.e 1, (1M, 4F) | N/A | Isolated optic atrophy | N/A | c.5A>C, p.Glu2Ala | Exon 1, GTPase | Heterozygous | [15] | |
| P.e 2, (6M, 2F) | N/A | Isolated optic atrophy | N/A | c.5A>C, p.Glu2Ala | Exon 1, GTPase | Heterozygous | [15] | |
| P.t 3  (F) | 1.2 years | DD, hypotonia, ataxia, dysarthria, microcephaly, nystagmus, scoliosis, optic atrophy, increased lactate, increased very-long-chain fatty acids | Alive,  7 years | c.95G>C, p.Gly32Ala | Exon 1, GTPase | *De novo* | [18] | |
| P.t 4  (M) | 1 year | DD, strabismus, ataxia, progressive infantile encephalopathy, dysarthria, brain MRI abnormal, increased lactate | Alive,  16 years | c.106A>G, p.Ser36Gly &c.346_347delGA, p.Glu116Lysfs*6 | Exon 2 & 4, GTPase | Compound heterozygous | [19] | |
| P.t 5  (M) | 1 year | DD, strabismus, dysmetria, dysarthria, progressive infantile encephalopathy, diffuse hyper-reflexia | Alive,  3 years | c.106A>G, p.Ser36Gly &c.346_347delGA, p.Glu116Lysfs*6 | Exon 2 & 4, GTPase | Compound heterozygous | [19] | |
| P.t 6  (M) | 5 months | DD, dystonia, GTCS, peripheral neuropathy,  EEG abnormal | Alive,  10 years | c.115A>G, p.Ser39Gly | Exon 2, GTPase | *De novo* | [20] | |
| P.t 7  (M) | 1 month | Psychomotor retardation, dystonia, microcephaly, brain MRI abnormal, hyperlactacidemia | Alive,  3 years | c.116G>A, p.Ser39Asn | Exon 2, GTPase | *De novo* | [21] | |
| P.t 8  (F) | 1 month | DD, GTCS, optic atrophy, ataxia, dysarthria, dystonia, nystagmus, scoliosis, pain insensitivity, peripheral neuropathy, increased lactate | Alive,  32 years | c.176C>A, p.Thr59Asn | Exon 2, GTPase | *De novo* | [22] | |
| P.t 9  (F) | Birth | Hypotonia, low birth weight,  peripheral neuropathy | Died, 3 weeks | c.261dup, p.Trp88Metfs* &c.385_386del, p.Glu129Lys*6 | Exon 3 & 5, GTPase | Compound heterozygous | [23] | |
| P.t 10  (M) | Birth | Hypotonia, low birth weight, brain MRI abnormal, peripheral neuropathy | Died, 8 days | c.261dup, p.Trp88Metfs* &c.385_386del, p.Glu129Lys*6 | Exon 3 & 5, GTPase | Compound heterozygous | [23] | |
| P.t 11  (F) | Infant | DD, dystonia, pain insensitivity,  brain MRI abnormal, increased lactate | Alive,  4 years | c.305C>T, p.Thr115Met | Exon 4,  GTPase | Homozygous | [5] | |
| P.t 12  (F) | 1 week | DD, hypotonia, ataxia, peripheral neuropathy, absent deep tendon reflexes, increased lactate | Alive,  5 years | c.436G>A, p.Asp146Asn | Exon 5,  GTPase | *De novo* | [24] | |
| P.t 13  (M) | 3 months | DD, ataxia, dysarthria, absent deep tendon reflexes, peripheral neuropathy | Alive,  4.5 years | c.445G>A,  p.Gly149Arg | Exon 5,  GTPase | *De novo* | [25] | |
| P.e 14, (2M, 1F) | N/A | Isolated optic atrophy | N/A | c.575C>A, p.Ala192Glu | Exon 6, GTPase | Heterozygous | [15] | |
| P.t 15  (F) | 2.5 years | GTCS, SRSE, ataxic, brain MRI abnormal,  EEG abnormal | Alive,  6 years | c.668G>T, p.Gly223Val | Exon 7,  GTPase | *De novo* | [9] | |
| P.t 16  (F) | 6 years | Epilepsy, ataxia, dystonia,  peripheral neuropathy | Died, 20 years | c.687_689dupATT, p.Leu230dup | Exon 7, GTPase | *De novo* | [8] | |
| P.t 17  (M) | 5 months | DD, epilepsy, nystagmus, hypotonia,  brain MRI abnormal, increased lactate | Died, 5 years | c.1048G>A, p.Gly350Arg | Exon 9, Middle | Heterozygous | [26] | |
| P.t 18  (M) | Birth | DD, pain insensitivity, dystonia, microcephaly, brain MRI abnormal, increased lactate | Alive,  2 years | c.1084G>A, p.Gly362Ser | Exon 10, Middle | *De novo* | [27] | |
| P.t 19  (M) | 4 months | DD, GTCS, RSE, dystonia, scoliosis,  pain insensitivity, brain MRI abnormal,  EEG abnormal, increased lactate | Died, 10 years | c.1084G>A, p.Gly362Ser | Exon 10, Middle | *De novo* | [28] | |
| P.t 20  (F) | 4 months | DD, epilepsy, microcephaly, dystonia,  pain insensitivity, dysphagia | Alive,  5 years | c.1084G>A, p.Gly362Ser | Exon 10, Middle | *De novo* | [9] | |
| P.t 21  (M) | 6 months | DD, GTCS, RSE, strabismus,  EEG abnormal | Alive,  7 years | c.1085G>A, p.Gly362Asp | Exon 10,  Middle | *De novo* | [29] | |
| P.t 22  (M) | 1 month | DD, SRSE, peripheral neuropathy,  brain MRI abnormal, EEG abnormal,  increased lactate | Died, 6 years | c.1085G>A,  p.Gly362Asp &c.1535T>C p.Ile512Thr | Exon 10 & 13, Middle & VD | Homozygous | [9] | |
| P.t 23  (F) | Birth | DD, epilepsy, microcephaly, hypotonia,  brain MRI abnormal, increased lactate,  ECG and echocardiogram abnormal | Died, 13 months | c.1088G>A, p.Gly363Asp | Exon 10, Middle | *De novo* | [8] | |
| P.t 24  (F) | 5 years | SRSE, brain MRI abnormal,  EEG abnormal | Alive,  7 years | c.1109T>G, p.Phe370Cys | Exon 10, Middle | *De novo* | [9] | |
| P.t 25  (F) | 4 days | DD, microcephaly, dystonia, brain MRI abnormal, persistent lactic acidosis | Died, 10 months | c.1135G>A, p.Glu379Lys | Exon 10, Middle | *De novo* | [26] | |
| P.t 26  (F) | 6 days | DD, microcephaly, optic atrophy, nystagmus, dystonia, brain MRI abnormal, increased very-long-chain fatty acids, increased lactate | Died, 37 days | c.1184C>A, p.Ala395Asp | Exon 10, Middle | *De novo* | [10] | |
| P.t 27  (F) | 6 months | DD, astigmatism, autistic features,  brain MRI abnormal | Alive,  10 years | c.1184C>G p.Ala395Gly | Exon 10, Middle | *De novo* | [18] | |
| P.t 28  (F) | 8 months | DD, epilepsy, microcephaly, ECG abnormal, dilated cardiomyopathy, increased lactate | Died, 10 months | c.1201G>A, p.Gly401Ser | Exon 11, Middle | *De novo* | [8] | |
| P.t 29  (M) | 2.8 years | DD, hypotonia, epilepsy, nystagmus, encephalopathy | Alive,  3 years | c.1201G>A, p.Gly401Ser | Exon 11, Middle | *De novo* | [8] | |
| P.t 30  (M) | 7 years | GTCS, dystonia, brain MRI abnormal | Died, 7 years | c.1207C>T, p.Arg403Cys | Exon 11, Middle | *De novo* | [30] | |
| P.t 31  (M) | 5 years | GTCS, FSE, dystonia, pain insensitivity,  brain MRI abnormal, EEG abnormal | Died, 7 years | c.1207C>T, p.Arg403Cys | Exon 11, Middle | *De novo* | [30] | |
| P.t 32  (F) | 3.4 years | DD, FSE, dystonia, hyperpyrexia,  brain MRI abnormal, EEG abnormal | Died, 3.7 years | c.1207C>T, p.Arg403Cys | Exon 11, Middle | *De novo* | [30] | |
| P.t 33  (F) | 3 years | Epilepsy, dystonia, brain MRI abnormal,  EEG abnormal, increased lactate | Alive,  ND | c.1207C>T, p.Arg403Cys | Exon 11, Middle | *De novo* | [31] | |
| P.t 34  (F) | 7 years | DD, RSE, ataxic, dysmetria,  brain MRI abnormal, EEG abnormal | Alive,  12 years | c.1207C>T, p.Arg403Cys | Exon 11, Middle | *De novo* | [32] | |
| P.t 35  (M) | 4 years | FSE, DR, dysphasia, encephalopathy,  brain MRI abnormal, EEG abnormal | Alive,  8 years | c.1207C>T, p.Arg403Cys | Exon 11, Middle | *De novo* | [13] | |
| P.t 36  (M) | 5 years | FSE, DR, dysarthria, encephalopathy,  brain MRI abnormal, EEG abnormal | Alive,  7 years | c.1207C>T, p.Arg403Cys | Exon 11, Middle | *De novo* | [13] | |
| P.t 37  (M) | 3 years | DD, FSE, brain MRI abnormal,  EEG abnormal, increased lactate | Alive,  ND | c.1207C>T,  p.Arg403Cys | Exon 11, Middle | *De novo* | [33] | |
| P.t 38  (M) | 5 years | DD, GTCS, FSE, ataxia, brain MRI abnormal, EEG abnormal, increased lactate | Alive,  ND | c.1207C>T, p.Arg403Cys | Exon 11, Middle | *De novo* | [33] | |
| P.t 39  (M) | 3 years | DD, GTCS, RSE, encephalopathy,  brain MRI abnormal, EEG abnormal | Alive,  ND | c.1207C>T,  p.Arg403Cys | Exon 11, Middle | *De novo* | [34] | |
| P.t 40  (F) | 9 years | FSE, DR, nystagmus, encephalopathy,  brain MRI abnormal, EEG abnormal | Died, 16 years | c.1207C>T, p.Arg403Cys | Exon 11, Middle | *De novo* | [12] | |
| P.t 41  (M) | 3 years | DD, SRSE, brain MRI abnormal,  EEG abnormal, increased lactate | Alive,  7 years | c.1207C>T, p.Arg403Cys | Exon 11, Middle | *De novo* | [9] | |
| P.t 42  (M) | 13 years | SRSE, progressive aphasia,  brain MRI abnormal, EEG abnormal | Died, 13.5 years | c.1207C>T, p.Arg403Cys | Exon 11, Middle | *De novo* | [35] | |
| P.t 43  (F) | 6 years | FSE, SRSE, excessive drooling,  brain MRI abnormal, EEG abnormal | Alive,  20 years | c.1207C>T, p.Arg403Cys | Exon 11, Middle | *De novo* | [40] | |
| P.t 44  (M) | 1.5 years | DD, GTCS, SRSE, dystonia,  EEG abnormal | Alive,  13 years | c.1207C>T, p.Arg403Cys | Exon 11, Middle | *De novo* | [36] | |
| P.t 45  (F) | 2 years | DD, DR, GTCS, dystonia, ataxia,  brain MRI abnormal | Alive,  6 years | c.1207C>T, p.Arg403Cys | Exon 11, Middle | ND | [18] | |
| P.t 46  (M) | 3 years | DD, DR, RSE, dysphagia, strabismus,  brain MRI abnormal, increased lactate | Alive,  7 years | c.1207C>T, p.Arg403Cys | Exon 11, Middle | *De novo* | [18] | |
| P.t 47  (M) | 6 months | DD, GTCS, hypotonia, nystagmus,  brain MRI abnormal, EEG abnormal,  increased lactate | Died, 1.5 year | c.1217T>C, p.Leu406Ser | Exon 11, Middle | *De novo* | [37] | |
| P.t 48  (F) | 4 months | DD, microcephaly, failure to thrive, hypotonia, echocardiogram abnormal, increased lactate | Died, 8 months | c. 1228G>A, p.Glu410Lys | Exon 11, Middle | *De novo* | [38] | |
| P.t 49  (F) | Birth | DD, DR, epilepsy, dysphagia, hypotonia,  brain MRI abnormal, EEG abnormal | Died, 1 year | c.1292G>A, p.Cys431Tyr | Exon 11, Middle | *De novo* | [18] | |
| P.t 50  (F) | 3 months | Regression and global encephalopathy,  brain MRI abnormal, hyperlactacidemia | Died, 2.5 years | c.1337G>T, p.Cys446Phe | Exon 11, Middle | *De novo* | [14] | |
| P.t 51  (F) | 3 months | DD, static encephalopathy, epilepsy,  hypotonia, nystagmus, scoliosis, optic atrophy, brain MRI abnormal | Alive,  27 years | c.2072A>G,  p.Tyr691Cys | Exon 19, GED | *De novo* | [39] | |
| P.t 52  (M) | 3 years | DR, epilepsy, optic atrophy, scoliosis,  movement disorder, peripheral neuropathy | Died, 17 years | c.2128A>G, p.Arg710Gly | Exon 19, GED | *De novo* | [8] | |
| P.t 53  (F) | 1.5 years | Hemiplegia, astigmatism, strabismus, paroxysmal limb dysfunction, increased acetoacetic acid | Alive,  5.3 years | c. 2161C>T, p.Gln721Ter | Exon 20, GED | *De novo* | Ours | |
| Abbreviations: DD = developmental delay; DR = developmental regression; GTCS = generalized tonic-clonic seizures; SRSE = super refractory status epilepticus; RSE = refractory status epilepticus; FSE = focal status epilepticus; VD = variable domain; GED = GTPase effector domain; P.e = pedigree; P.t = patient; M = male; F = female; N/A = not applicable; ND = no description. | | | | | | | | |

**References**

5. Hogarth KA, Costford SR, Yoon G, Sondheimer N, Maynes JT: **DNM1L Variant Alters Baseline Mitochondrial Function and Response to Stress in a Patient with Severe Neurological Dysfunction**. *Biochemical genetics* 2018, **56**(1-2):56-77.

8. Nolden KA, Egner JM, Collier JJ, Russell OM, Alston CL, Harwig MC, Widlansky ME, Sasorith S, Barbosa IA, Douglas AG *et al*: **Novel &lt;i&gt;DNM1L&lt;/i&gt; variants impair mitochondrial dynamics through divergent mechanisms**. *Life science alliance* 2022, **5**(12).

9. Verrigni D, Di Nottia M, Ardissone A, Baruffini E, Nasca A, Legati A, Bellacchio E, Fagiolari G, Martinelli D, Fusco L *et al*: **Clinical-genetic features and peculiar muscle histopathology in infantile DNM1L-related mitochondrial epileptic encephalopathy**. *Human mutation* 2019, **40**(5):601-618.

10. Waterham HR, Koster J, van Roermund CW, Mooyer PA, Wanders RJ, Leonard JV: **A lethal defect of mitochondrial and peroxisomal fission**. *The New England journal of medicine* 2007, **356**(17):1736-1741.

12. Nolan DA, Chen B, Michon AM, Salatka E, Arndt D: **A Rasmussen encephalitis, autoimmune encephalitis, and mitochondrial disease mimicker: expanding the DNM1L-associated intractable epilepsy and encephalopathy phenotype**. *Epileptic disorders : international epilepsy journal with videotape* 2019, **21**(1):112-116.

13. Fahrner JA, Liu R, Perry MS, Klein J, Chan DC: **A novel de novo dominant negative mutation in DNM1L impairs mitochondrial fission and presents as childhood epileptic encephalopathy**. *American journal of medical genetics Part A* 2016, **170**(8):2002-2011.

14. Díez H, Cortès-Saladelafont E, Ormazábal A, Marmiese AF, Armstrong J, Matalonga L, Bravo M, Briones P, Emperador S, Montoya J *et al*: **Severe infantile parkinsonism because of a de novo mutation on DLP1 mitochondrial-peroxisomal protein**. *Movement disorders : official journal of the Movement Disorder Society* 2017, **32**(7):1108-1110.

15. Gerber S, Charif M, Chevrollier A, Chaumette T, Angebault C, Kane MS, Paris A, Alban J, Quiles M, Delettre C *et al*: **Mutations in DNM1L, as in OPA1, result in dominant optic atrophy despite opposite effects on mitochondrial fusion and fission**. *Brain : a journal of neurology* 2017, **140**(10):2586-2596.

18. Whitley BN, Lam C, Cui H, Haude K, Bai R, Escobar L, Hamilton A, Brady L, Tarnopolsky MA, Dengle L *et al*: **Aberrant Drp1-mediated mitochondrial division presents in humans with variable outcomes**. *Human molecular genetics* 2018, **27**(21):3710-3719.

19. Nasca A, Legati A, Baruffini E, Nolli C, Moroni I, Ardissone A, Goffrini P, Ghezzi D: **Biallelic Mutations in DNM1L are Associated with a Slowly Progressive Infantile Encephalopathy**. *Human mutation* 2016, **37**(9):898-903.

20. Keller N, Paketci C, Edem P, Thiele H, Yis U, Wirth B, Karakaya M: **De novo DNM1L variant presenting with severe muscular atrophy, dystonia and sensory neuropathy**. *European journal of medical genetics* 2021, **64**(2):104134.

21. Liu X, Zhang Z, Li D, Lei M, Li Q, Liu X, Zhang P: **DNM1L-Related Mitochondrial Fission Defects Presenting as Encephalopathy: A Case Report and Literature Review**. *Frontiers in pediatrics* 2021, **9**:626657.

22. Lhuissier C, Wagner BE, Vincent A, Garraux G, Hougrand O, Van Coster R, Benoit V, Karadurmus D, Lenaers G, Gueguen N *et al*: **Case report: Thirty-year progression of an EMPF1 encephalopathy due to defective mitochondrial and peroxisomal fission caused by a novel de novo heterozygous DNM1L variant**. *Frontiers in neurology* 2022, **13**:937885.

23. Yoon G, Malam Z, Paton T, Marshall CR, Hyatt E, Ivakine Z, Scherer SW, Lee KS, Hawkins C, Cohn RD: **Lethal Disorder of Mitochondrial Fission Caused by Mutations in DNM1L**. *The Journal of pediatrics* 2016, **171**:313-316.e311-312.

24. Longo F, Benedetti S, Zambon AA, Sora MGN, Di Resta C, De Ritis D, Quattrini A, Maltecca F, Ferrari M, Previtali SC: **Impaired turnover of hyperfused mitochondria in severe axonal neuropathy due to a novel DRP1 mutation**. *Human molecular genetics* 2020, **29**(2):177-188.

25. Wei Y, Qian M: **Case Report: A Novel de novo Mutation in DNM1L Presenting With Developmental Delay, Ataxia, and Peripheral Neuropathy**. *Frontiers in pediatrics* 2021, **9**:604105.

26. Chao YH, Robak LA, Xia F, Koenig MK, Adesina A, Bacino CA, Scaglia F, Bellen HJ, Wangler MF: **Missense variants in the middle domain of DNM1L in cases of infantile encephalopathy alter peroxisomes and mitochondria when assayed in Drosophila**. *Human molecular genetics* 2016, **25**(9):1846-1856.

27. Sheffer R, Douiev L, Edvardson S, Shaag A, Tamimi K, Soiferman D, Meiner V, Saada A: **Postnatal microcephaly and pain insensitivity due to a de novo heterozygous DNM1L mutation causing impaired mitochondrial fission and function**. *American journal of medical genetics Part A* 2016, **170**(6):1603-1607.

28. Tarailo-Graovac M, Zahir FR, Zivkovic I, Moksa M, Selby K, Sinha S, Nislow C, Stockler-Ipsiroglu SG, Sheffer R, Saada-Reisch A *et al*: **De novo pathogenic DNM1L variant in a patient diagnosed with atypical hereditary sensory and autonomic neuropathy**. *Molecular genetics & genomic medicine* 2019, **7**(10):e00961.

29. Vanstone JR, Smith AM, McBride S, Naas T, Holcik M, Antoun G, Harper ME, Michaud J, Sell E, Chakraborty P *et al*: **DNM1L-related mitochondrial fission defect presenting as refractory epilepsy**. *European journal of human genetics : EJHG* 2016, **24**(7):1084-1088.

30. Pan Z, Wu TH, Chen C, Peng P, He YW, Yi WZ, Yin F, Peng J: **[DNM1L gene variant caused encephalopathy, lethal, due to defective mitochondrial peroxisomal fission 1: three cases report and literature review]**. *Zhonghua Er Ke Za Zhi* 2021, **59**(5):400-406.

31. Chen X, Li Y, Luo H, Gan J: **[Analysis of DNM1L gene variant in a case of fatal encephalopathy caused by mitochondrial peroxidase division deficiency]**. *Zhonghua yi xue yi chuan xue za zhi = Zhonghua yixue yichuanxue zazhi = Chinese journal of medical genetics* 2021, **38**(9):887-890.

32. De Souza Crippa AC, Franklin GL, Takeshita BT, Ghizoni Teive HA: **DNM1L mutation presenting as progressive myoclonic epilepsy associated with acute febrile infection-related epilepsy syndrome**. *Epileptic disorders : international epilepsy journal with videotape* 2022, **24**(5):976-978.

33. Ladds E, Whitney A, Dombi E, Hofer M, Anand G, Harrison V, Fratter C, Carver J, Barbosa IA, Simpson M *et al*: **De novo DNM1L mutation associated with mitochondrial epilepsy syndrome with fever sensitivity**. *Neurology Genetics* 2018, **4**(4):e258.

34. Schmid SJ, Wagner M, Goetz C, Makowski C, Freisinger P, Berweck S, Mall V, Burdach S, Juenger H: **A De Novo Dominant Negative Mutation in DNM1L Causes Sudden Onset Status Epilepticus with Subsequent Epileptic Encephalopathy**. *Neuropediatrics* 2019, **50**(3):197-201.

35. Mancardi MM, Nesti C, Febbo F, Cordani R, Siri L, Nobili L, Lampugnani E, Giacomini T, Granata T, Marucci G *et al*: **Focal status and acute encephalopathy in a 13-year-old boy with de novo DNM1L mutation: Video-polygraphic pattern and clues for differential diagnosis**. *Brain Dev* 2021, **43**(5):644-651.

36. Ryan CS, Fine AL, Cohen AL, Schiltz BM, Renaud DL, Wirrell EC, Patterson MC, Boczek NJ, Liu R, Babovic-Vuksanovic D *et al*: **De Novo DNM1L Variant in a Teenager With Progressive Paroxysmal Dystonia and Lethal Super-refractory Myoclonic Status Epilepticus**. *J Child Neurol* 2018, **33**(10):651-658.

37. Zaha K, Matsumoto H, Itoh M, Saitsu H, Kato K, Kato M, Ogata S, Murayama K, Kishita Y, Mizuno Y *et al*: **DNM1L-related encephalopathy in infancy with Leigh syndrome-like phenotype and suppression-burst**. *Clinical genetics* 2016, **90**(5):472-474.

38. Vandeleur D, Chen CV, Huang EJ, Connolly AJ, Sanchez H, Moon-Grady AJ: **Novel and lethal case of cardiac involvement in DNM1L mitochondrial encephalopathy**. *American journal of medical genetics Part A* 2019, **179**(12):2486-2489.

39. Assia Batzir N, Bhagwat PK, Eble TN, Liu P, Eng CM, Elsea SH, Robak LA, Scaglia F, Goldman AM, Dhar SU *et al*: **De novo missense variant in the GTPase effector domain (GED) of DNM1L leads to static encephalopathy and seizures**. *Molecular Case Studies* 2019, **5**(3).

40. Minghetti S, Giorda R, Mastrangelo M, Tassi L, Zanotta N, Galbiati S, Bassi MT, Zucca C: **Epilepsia partialis continua associated with the p.Arg403Cys variant of the DNM1L gene: an unusual clinical progression with two episodes of super-refractory status epilepticus with a 13-year remission interval**. *Epileptic disorders : international epilepsy journal with videotape* 2022, **24**(1):176-182.
